# Genome-wide association study identifies QTL for eight fruit traits in cultivated tomato (*Solanum lycopersicum* L.)

**DOI:** 10.1038/s41438-021-00638-4

**Published:** 2021-09-01

**Authors:** Minkyung Kim, Thuy Tien Phan Nguyen, Joon-Hyung Ahn, Gi-Jun Kim, Sung-Chur Sim

**Affiliations:** 1 Department of Bioresources Engineering, Sejong University, Seoul, Republic of Korea; 2 Asia Seed R&D center, Icheon, Republic of Korea; 3 Plant Engineering Research Institute, Sejong University, Seoul, Republic of Korea

**Keywords:** Plant breeding, Agricultural genetics

## Abstract

Genome-wide association study (GWAS) is effective in identifying favorable alleles for traits of interest with high mapping resolution in crop species. In this study, we conducted GWAS to explore quantitative trait loci (QTL) for eight fruit traits using 162 tomato accessions with diverse genetic backgrounds. The eight traits included fruit weight, fruit width, fruit height, fruit shape index, pericarp thickness, locule number, fruit firmness, and brix. Phenotypic variations of these traits in the tomato collection were evaluated with three replicates in field trials over three years. We filtered 34,550 confident SNPs from the 51 K Axiom^®^ tomato array based on < 10% of missing data and > 5% of minor allele frequency for association analysis. The 162 tomato accessions were divided into seven clusters and their membership coefficients were used to account for population structure along with a kinship matrix. To identify marker-trait associations (MTAs), four phenotypic data sets representing each of three years and combined were independently analyzed in the multilocus mixed model (MLMM). A total of 30 significant MTAs was detected over data sets for eight fruit traits at *P* < 0.0005. The number of MTA per trait ranged from one (brix) to seven (fruit weight and fruit width). Two SNP markers on chromosomes 1 and 2 were significantly associated with multiple traits, suggesting pleiotropic effects of QTL. Furthermore, 16 of 30 MTAs suggest potential novel QTL for eight fruit traits. These results facilitate genetic dissection of tomato fruit traits and provide a useful resource to develop molecular tools for improving fruit traits via marker-assisted selection and genomic selection in tomato breeding programs.

## Introduction

Tomato (*Solanum lycopersicum* L.) is an economically important crop species in the Solanaceae family, which includes potato, pepper, and eggplant. It is cultivated worldwide and one of the most consumed vegetables. In 2018, the world production of tomato exceeded 182 million tons from 4.76 million ha^1^. With its economic value, large efforts have been made to improve horticultural traits and disease resistance in tomato breeding programs. Tomato has diverse genetic variations in fruit traits, such as shape, size, and weight. Therefore, QTL mapping has been extensively conducted using bi-parental populations for genetic dissection of fruit traits and several major genes were identified^2–7^. QTL detection in the structured populations derived from two parents has the disadvantage of low mapping resolution due to limited recombination events^8,9^.

As an effective mapping method for complex traits, genome-wide association study (GWAS) allows to identify the tight linkage between marker and QTL with dense genome coverage in the unstructured populations, such as collections of germplasm and breeding lines. These GWAS panels have higher recombination rates to increase mapping resolutions relative to bi-parental populations^10^. In addition, diverse alleles for a trait of interest can be explored in these populations^11,12^. The discovery of genome-wide single nucleotide polymorphisms (SNPs) has facilitated GWAS in crop species. As the most common type of sequence variation, SNPs are suitable for high-throughput genotyping with automation. Advances in next-generation sequencing (NGS) technology have led to an accumulation of SNPs. In tomato, a NGS-based transcriptome analysis of five cultivated varieties and one wild species generated 17 Gb of sequences and identified 62,576 non-redundant SNPs^13^. Of these, 8784 SNPs were used to develop the first high-throughput genotyping array^14^. Whole-genome sequencing of diverse tomato accessions also identified a large number of SNPs across 12 chromosomes^15,16^. Furthermore, a total of 51,912 SNPs was detected by resequencing 96 large-fruit commercial varieties with a mean depth of 1.9x and these SNPs were used to develop the Axiom tomato genotyping array^17^.

In addition to NGS-based SNP discovery, several statistical models have been developed to improve the accuracy and efficiency of GWAS^10,18,19^. With these advances, GWAS has been successfully conducted to explore allele variations for fruit quality and morphology in tomato. Several marker-trait associations (MTAs) were detected for phenolic compounds, ascorbic acid, β-carotene, trans-lycopene, and titratable acidity using the worldwide collection of 96 accessions representing landraces, vintage, and modern varieties^20^. GWAS in 163 tomato accessions identified a total of 44 candidate loci for 19 fruit metabolites, including amino acids, sucrose, malate, ascorbate, and citrate^21^. Two mapping populations were also used to investigate the genetic architecture of tocochromanol content in tomato fruit^22^. Genetic dissection of tomato flavor was also conducted and a large number of significant associations was found for flavor-related traits^23–25^. For fruit morphological traits, a number of favorable alleles was detected by GWAS in the tomato collections^26,27^. A recent study investigated genetic variations for six fruit traits in 192 tomato accessions and identified a total of 54 loci associated with these traits^28^. In addition, a germplasm collection of 163 accessions representing *S. lycopersicum* and *S. pimpinellifolium* was used to identify genomic regions associated with fruit, flower, and vegetative traits via GWAS^29^. This study revealed a total of 107 MTAs for eight quantitative traits, including fruit weight and locule number.

Although a number of loci associated with fruit traits was found in the previous studies, these loci are responsible for partial genetic variations of each trait in tomato. Therefore, the present study was conducted to explore novel QTL for eight fruit traits in a collection of 162 tomato accessions representing different genetic backgrounds from the previous GWAS panels. The eight fruit traits used in our study included fruit weight, fruit width, fruit height, fruit shape index, pericarp thickness, locule number, fruit firmness, and brix. GWAS with phenotypic data from field trials over three years identified a number of potential novel QTL along with previously known genes. These results will be a useful resource to develop breeder’s toolboxes for marker-assisted selection and genomic selection in tomato breeding programs.

## Results

### Genome-wide SNP identification

The 51,214 SNPs of the Axiom^®^ tomato array were polymorphic in the 162 tomato accessions. Of these, 34,550 SNPs were filtered with missing data rate (< 10%) and minor allele frequency (> 5%). These confident SNPs were distributed over 12 chromosomes and covered a total of 751.75 Mb with a range of 45.52 Mb on chromosome 6–90.24 Mb on chromosome 1 (Table 1). The number of SNPs per chromosome ranged from 1292 (chromosome 7) to 5469 (chromosome 1). In addition, the average of marker intervals across all 12 chromosomes was 0.021 Mb, ranging from 0.013 Mb on chromosome 11 to 0.050 Mb on chromosome 7 (Table 1). The largest gap of 21.60 Mb was found on chromosome 9, while the maximum marker intervals were 1.05–15.71 Mb on the other chromosomes.

**Table 1:** Distribution of 34,550 confident SNP markers on 12 tomato chromosomes

Chromosome	No. of SNP markers	Coverage (Mb)^a^	Marker interval (Mb)	
			Maximum	Average
1	5469	90.24	7.12	0.016
2	2211	49.37	1.58	0.022
3	2942	62.54	2.33	0.021
4	3665	63.87	15.71	0.017
5	2017	63.95	4.96	0.031
6	3435	45.52	2.54	0.013
7	1292	64.86	2.65	0.050
8	1491	62.82	2.22	0.042
9	3838	67.65	21.60	0.017
10	1453	64.81	2.89	0.044
11	3816	50.84	12.47	0.013
12	2921	65.28	1.05	0.022
Total	34,550	751.75	21.60	0.021

^a^Coverage was determined using the tomato genome assembly SL4.0

### Phenotypic variations of fruit traits in the tomato collection

**Fig. 1:**
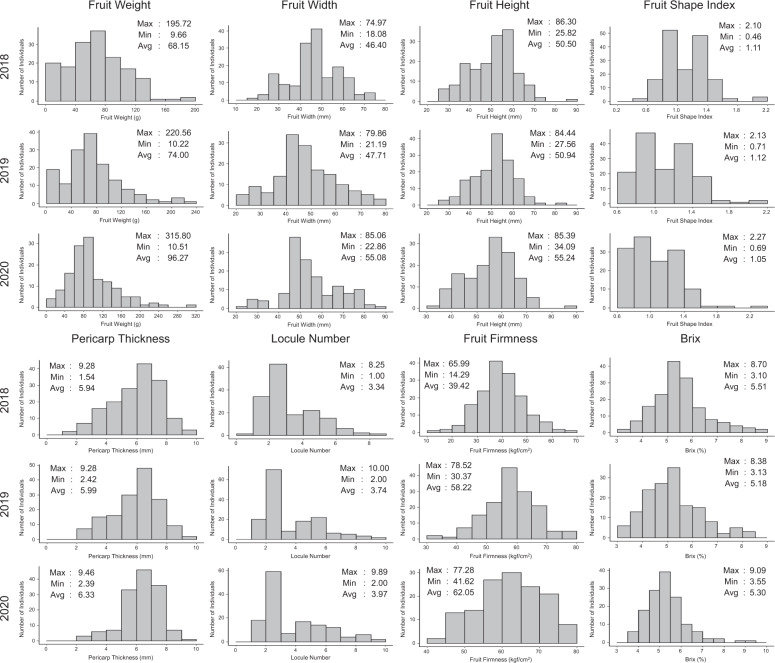
Phenotypic distribution of eight fruit traits in the 162 tomato accessions over three years. Max: maximum value, Min: minimum value, and Avg: mean value in the tomato collection

The 162 tomato accessions showed wide ranges of phenotypic variations for eight fruit traits, including fruit weight, fruit width, fruit height, fruit shape index, pericarp thickness, locule number, fruit firmness, and brix (Figs. 1 and S1). Fruit weight ranged from 9.66 to 315.80 g, and the means of each year were 68.15 g in 2018, 74.00 g in 2019, and 96.27 g in 2020. The Pearson correlation coefficients between the three years were 0.75–0.85 (Table 2). We found phenotypic variations of 18.08–85.06 mm for fruit width and 25.82–86.30 mm for fruit height. These traits also showed high levels of correlation over three years with coefficients of 0.78–0.86 for width and 0.78–0.83 for height. Fruit shape index showed the highest correlation ranging of 0.93–0.95 in 2018–2020 with the phenotypic variations of 0.46–2.27 with means of 1.05–1.12 (Fig. 1 and Table 2). These results indicate that the tomato collection represents diverse fruit sizes and shapes.

**Table 2:** Phenotypic correlation of eight fruit traits in the 162 tomato accessions over three years

Trait	Pearson correlation coefficient		
	2018 vs. 2019	2018 vs. 2020	2019 vs. 2020
Fruit weight (g)	0.85	0.75	0.76
Fruit width (mm)	0.85	0.78	0.86
Fruit height (mm)	0.83	0.78	0.80
Fruit shape index	0.94	0.93	0.95
Pericarp thickness (mm)	0.85	0.76	0.77
Locule number	0.83	0.84	0.88
Fruit firmness (kgf/cm^2^)	0.33	0.33	0.81
Brix (%)	0.72	0.45	0.52

For the other traits, substantial phenotypic variations were observed for pericarp thickness and locule number with the correlation coefficients of 0.76–0.85 and 0.83–0.88 over three years (Fig. 1 and Table 2). Fruit firmness showed the means of 39.42 kgf/cm^2^ in 2018, 58.22 kgf/cm^2^ in 2019, and 62.05 kgf/cm^2^ in 2020. In addition, the 2018 phenotypic data of this trait showed a low correlation coefficient of 0.33 with each of the 2019 and 2020 data relative to the coefficient of 0.81 between 2019 and 2020 (Table 2). This is due to the difference between destructive (2018) and non-destructive (2019 and 2020) methods. Brix ranged from 3.10 to 9.09% over three years with the means of 5.18–5.51% and correlation coefficients of 0.45–0.72 (Fig. 1 and Table 2). The phenotypic data of three years for eight fruit traits were used for GWAS without normalization.

### Identification of marker-trait associations for fruit traits

**Fig. 2:**
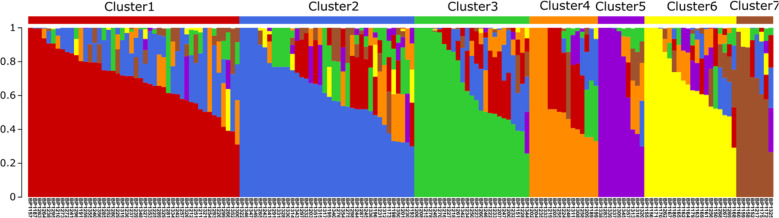
Inferred population structure in the 162 tomato accessions using the model-based program STRUCTURE v2.3.4. A single vertical line indicates each accession’s genome, which is partitioned into colored segments in proportion to the estimated membership in the seven clusters

The 34,550 confident SNPs were used to infer a population structure in the 162 tomato accessions representing 29 small fruit (round, cylinder, and oval), 119 medium fruit (flat, cylinder, oval, and round), and 14 large fruit (flat) germplasm. The delta K method^30^ suggested that the best K (number of clusters) was seven in the model-based clustering analysis and the number of tomato accessions per cluster ranged from eight (cluster 7) to 46 (cluster 1) (Fig. 2 and Table S1). Cluster 1, which is the largest, consisted of 42 medium fruit accessions and four large fruit accessions. Of these medium fruit accessions, the oval shape was dominant (20 accessions) followed by 11 cylinder, seven flat, and four round accessions. Cluster 2 included 32 medium fruit accessions (18 flat, seven round, five oval, and two cylinder accessions). Six large fruit accessions were also found with these medium fruit accessions in this cluster. The other 44 medium fruit accessions were divided into clusters 3 (23 accessions), 4 (14 accessions), and 5 (seven accessions). In these clusters, we also found two small fruit accessions (cluster 3), one large fruit accession (cluster 4), and three large fruit accessions (cluster 5). Cluster 6 consisted of only 20 small fruit accessions (17 cylinder, two oval, and one round). Similarly, the dominant accession in cluster 7 was small fruit accessions (five round and two oval plum). This cluster also included a medium flat fruit accession (Fig. 2 and Table S1). In addition, the hierarchical clustering analysis based on Nei’s genetic distance also found that most of the accessions were grouped as shown in the seven clusters (Fig. S2). Considering geographic relations, 104 of 162 tomato accessions were collected from India and distributed into six clusters excluding cluster 7 (Table S1). Furthermore, we found no country-specific clusters in the other 58 accessions. Semi-determinant and determinant accessions were found together in the same cluster. Therefore, the population structure in the tomato collection is likely due to multiple factors, such as fruit size and pedigree.

**Fig. 3:**
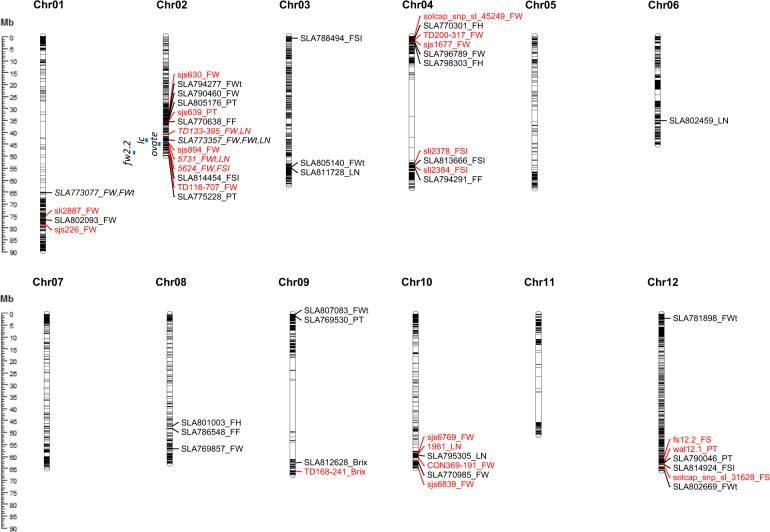
Physical map positions of the 30 marker-trait associations (MTAs) detected in this study and the previously known loci for eight fruit traits. The MTAs are prsented on the right side of chromosomes using SLAxxxxxx_trait name (FW: fruit weight, FWt: fruit width, FH: fruit height, FSI: fruit shape index, PT: pericarp thickness, LN: locule number, FF: fruit firmness, and brix). Two SNP markers (SLA773077 and SLA773357) associated with multiple traits are italicized. Three major genes (*fw2.2*, *lc*, and *ovate*) are shown on the left side of chromosome 2 and the MTAs reported in other studies are highlighted with a red color^2,7,27,28,32–35^

GWAS using the 34,550 confident SNPs identified a total of 30 significant marker-trait associations (MTAs) for eight fruit traits at *P* < 0.0005 (Table 3, Fig. 3, and S3). These MTAs were repeatedly detected in at least two of four phenotypic data sets (each of three years and combined). Of these, we found that 16 MTAs were significant at *P* < 0.00005 that was determined as a genome-wide significance threshold based on 1677 SNPs, the effective number of independent markers^31^. For fruit weight, seven MTAs were found on chromosomes 1, 2, 4, 8, and 10 at *P* < 0.0005, and five of these MTAs also showed significance at *P* < 0.00005 (Table 3 and Fig. 3). The phenotypic variance explained (PVE) for two MTAs on chromosomes 1 and 8 ranged from 11.98 to 28.95%, while the PVE for other MTAs were < 10%. In addition, two MTAs were detected within several Mb distances on both chromosomes 1 (11.46 Mb) and 2 (8.88 Mb). We found significant MTAs for fruit shape-related traits, fruit width (seven MTAs), fruit height (three MTAs), and fruit shape index (four MTAs) at *P* < 0.0005 (Table 3 and Fig. 3). Two MTAs for fruit width on chromosomes 1 and 2 explained 18.66–25.79% of phenotypic variance. Of these, the SLA773077 marker on chromosome 1 showed significant associations with both fruit weight and width. Furthermore, the 2nd MTA on chromosome 2 was found at 9.23 Mb away from the 1st MTA and its marker (SLA773357) was significantly associated with both the fruit weight and width (Table 3 and Fig. 3). These MTAs on chromosome 2 were also detected at *P* < 0.00005. For fruit height, one of two MTAs on chromosome 4 showed significance at *P* < 0.000005 and its PVE ranged from 20.27 to 42.60%. Another MTA was found on chromosome 8, explaining 15.44% (2018) and 9.82% (2019) of phenotypic variance. Three of four MTAs for fruit shape index were detected on chromosomes 2, 4, and 12 at *P* < 0.00005 (Table 3 and Fig. 3). The MTA on chromosome 2 showed large effects (up to 31.46% in the combined data), while the other MTAs explained < 10% of phenotypic variance.

**Table 3:** Significant associations for eight fruit traits identified repeatedly using the multilocus mixed model in the 162 tomato accessions

Trait	SNP^a^	Chr	Position^b^	*P* value				PVE (%)^c^			
				2018	2019	2020	Combined^d^	2018	2019	2020	Combined
Fruit weight	*SLA773077*	1	65.81	0.0000476	0.0179588	0.0006453	0.0002271	28.95	–		11.98
Fruit weight	SLA802093	1	77.27	0.0003554	0.0283558	0.0053169	0.0004674	7.77	–	–	3.07
Fruit weight	*SLA790460*	2	35.19	0.0001787	0.0000011	0.1634972	0.0001411	1.79	2.44	–	2.91
Fruit weight	*SLA773357*	2	44.07	0.0000108	0.0438821	0.0174063	0.0000190	5.82	–	–	5.97
Fruit weight	*SLA796789*	4	2.39	0.0050597	< 0.0000001	0.0190282	0.0000547	–	5.76	–	3.81
Fruit weight	*SLA769857*	8	56.89	0.0036812	< 0.0000001	0.0031018	0.0001049	–	28.21	–	26.20
Fruit weight	SLA770985	10	62.24	0.0040817	0.0015545	0.0002492	0.0001610	–	–	27.45	7.00
Fruit width	SLA773077	1	65.81	0.0023288	0.0003581	0.0000898	0.0000700	–	20.43	25.79	22.94
Fruit width	*SLA794277*	2	34.84	0.0000387	0.0069278	0.2426664	0.0000234	19.93	–	–	18.66
Fruit width	*SLA773357*	2	44.07	0.0000003	0.0002005	0.0004138	0.0000022	9.26	7.28	2.08	7.87
Fruit width	SLA805140	3	55.04	0.0002758	0.0512368	0.0033326	0.0004037	4.95	–	–	4.12
Fruit width	SLA807083	9	0.16	0.0041339	0.0001966	0.3589960	0.0001213	–	6.95	–	2.27
Fruit width	SLA781898	12	1.84	0.0022950	0.0001561	0.0214374	0.0001196	–	0.74	–	0.24
Fruit width	SLA802669	12	65.83	0.0027364	0.0003636	0.0009566	0.0000810	–	7.25	–	5.32
Fruit height	SLA770301	4	1.21	0.0103264	0.0002059	0.0004259	0.0001397	–	11.44	6.45	6.39
Fruit height	*SLA798303*	4	2.98	0.0005463	0.0002960	< 0.0000001	0.0000035	–	28.10	20.27	42.60
Fruit height	*SLA801003*	8	47.36	0.0000002	0.0002787	0.0048167	0.0008780	15.44	9.82	–	–
Fruit shape index	*SLA814454*	2	47.19	0.0000670	0.0108237	0.9941488	< 0.0000001	25.86	–	–	31.46
Fruit shape index	SLA788494	3	0.85	0.1903665	0.0000626	0.5070271	0.0003350	–	12.41	–	4.05
Fruit shape index	*SLA813666*	4	54.56	0.0000631	0.0021089	0.0248604	< 0.0000001	5.31	–	–	3.19
Fruit shape index	*SLA814924*	12	63.42	0.0000005	0.0169602	0.0110991	< 0.0000001	6.33	–	–	5.90
Pericarp thickness	SLA805176	2	35.89	0.0002759	0.0264039	0.0388244	0.0002181	1.94	–	–	2.08
Pericarp thickness	SLA775228	2	50.74	0.0013494	0.0004428	0.1007583	0.0002261	–	27.70	–	29.22
Pericarp thickness	*SLA769530*	9	0.89	0.0000279	0.0032031	0.0032080	0.0002705	1.04	–	–	0.38
Pericarp thickness	SLA790046	12	62.59	0.0153631	0.0004161	0.0165053	0.0004021	–	3.69	–	2.66
Locule number	*SLA773357*	2	44.07	< 0.0000001	0.0348780	0.2906442	0.0000009	12.05	–	–	10.47
Locule number	*SLA811728*	3	55.56	0.0003184	0.0007633	0.0035344	0.0000163	12.07	–	–	15.38
Locule number	SLA802459	6	36.01	0.0295386	0.0001617	0.0001102	0.0014632	–	1.55	5.22	–
Locule number	*SLA795305*	10	59.32	0.0001917	0.0000065	0.0030642	0.0025213	0.87	2.23	–	–
Fruit firmness	*SLA770638*	2	36.56	0.8059914	0.0004831	0.0000046	0.0000502	–	1.86	16.35	9.22
Fruit firmness	SLA794291	4	55.17	0.2954367	0.0001228	0.0034657	0.0000832	–	15.48	–	20.34
Fruit firmness	SLA786548	8	48.40	0.9845478	0.0001178	0.0005731	0.0001857	–	23.73	–	14.94
Brix	*SLA812628*	9	62.64	0.0000395	0.0148190	0.5575202	0.0000594	26.22	–	–	28.73

^a^SNP markers associated with the traits at *P* < 0.00005 were italicized

^b^The physical map positions of SNP markers were determined using the tomato genome assembly SL4.0

^c^The phenotypic variation explained (PVE) by each marker

^d^The phenotypic data of three years for seven traits were combined for GWAS. For fruit firmness, the 2019 and 2020 data were combined because the 2018 data were collected using a different type of penetrometer

We detected four MTAs for pericarp thickness on chromosomes 2, 9, and 12 at *P* < 0.0005 (Table 3 and Fig. 3). The SLA769530 marker on chromosome 9 was also associated with this trait at *P* < 0.00005 in the 2018 data. Two MTAs on chromosome 2 were found at 35.89 and 50.74 Mb, respectively. The 1st MTA explained up to 29.22% of phenotypic variance in the combined data. In contrast, the other MTAs including the 2nd MTA on chromosome 2 showed small effects (< 5%). For locule number, four MTAs were detected on chromosomes 2, 3, 6, and 10 at *P* < 0.0005, and three of these (excluding one on chromosome 6) also showed significance at *P* < 0.00005 (Table 3 and Fig. 3). Two of these MTAs on chromosomes 2 and 3 explained from 10.47 to 15.38% of phenotypic variance, while the other MTAs explained < 5%. In addition, the SLA773357 marker on chromosome 2 was significantly associated with not only locule number but also the fruit weight and width (Table 3 and Fig. 3). Association analysis with fruit firmness identified three MTAs on chromosomes 2, 4, and 8 at *P* < 0.0005 (Table 3 and Fig. 3). The PVE for two MTAs on chromosome 4 and 8 ranged from 14.94 to 23.73%. The MTA on chromosome 2 was significantly found at *P* < 0.000005 in the 2020 data and its PVE was 16.35%. For brix, only one significant MTA was detected on chromosome 9 at *P* < 0.00005 (2018) and *P* < 0.0005 (combined), explaining 26.22 and 28.73% of phenotypic variance in these data (Table 3 and Fig. 3).

## Discussion

In this study, a collection of 162 tomato accessions was used to identify favorable alleles associated with eight fruit traits using genome-wide SNPs. Their phenotypic variations of the traits were evaluated in field trials over three years. The observed large phenotypic variation of each trait in every year suggests that the tomato accessions originated from diverse genetic backgrounds. This genetic diversity provided an opportunity to explore novel QTL for improving the fruit traits in tomato breeding programs. Furthermore, six traits excluding fruit firmness and brix revealed high correlation coefficients (0.75–0.95) between the phenotypic data collected over three years. For fruit firmness, the 2018 phenotypic data showed the coefficient of 0.33 to each of the 2019 and 2020 data, while the coefficient between 2019 and 2020 was 0.81. We used a digital destructive penetrometer in 2018 and a non-destructive penetrometer in 2019 and 2020. Although both destructive and non-destructive penetrometers are commonly used to measure fruit firmness in crop species, our result demonstrates that these types of penetrometer can generate inconsistent measurements in tomato. Brix also showed relatively low correlation coefficients, especially when the phenotypic data in 2020 was compared with each of the 2018 and 2019 data. It is likely due to high precipitation and temperature in the 2020 growing season. In addition, this result is supported by lower heritability of brix (0.63) than other fruit traits, such as fruit weight (0.83) and locule number (0.85) reported in a previous study, indicating that this trait is more sensitive to environmental variations^7,36^.

For GWAS, we used the multilocus mixed model (MLMM) that effectively reduces false positives and false negatives^18^. A total of 30 significant MTAs was found with at least two phenotypic data sets for eight fruit traits at *P* < 0.0005. In addition, candidate genes for 10 MTAs were found using the tomato genome assembly SL4.0 and ITAG 4.0 (Table S2). Of the 30 MTAs, 14 likely represent previously known loci for fruit traits. There are three major genes for tomato fruit development on chromosome 2, including *fw2.2* for fruit weight at 50.29 Mb^37^, *ovate* for fruit shape at 46.38 Mb^38^, and *lc* for locule number at 45.19 Mb^4^. Two MTAs were detected in the genomic regions of these major genes on chromosome 2. One of these MTAs at 44.07 Mb showed significant associations with three traits (fruit weight, fruit width, and locule number), while another MTA was significant for the fruit shape index. We also found two additional MTAs for the fruit weight (35.19 Mb) and fruit width (34.84 Mb) in the known QTL region on chromosome 2^28^. The other three MTAs for fruit weight are likely to correspond to the known QTL on chromosomes 1, 4, and 10^28,33,39^. For the fruit shape index, two MTAs were found in the previously reported QTL regions on chromosomes 4 and 12^2,39^. Furthermore, two MTAs for pericarp thickness were found 0.45 Mb and 0.70 Mb away from the *lc* gene^4^ and known QTL^28^ for fruit weight on chromosome 2, respectively. A QTL for this trait was previously reported in the vicinity of our MTA on chromosome 12^2^. For brix, a single MTA was detected at 62.64 Mb on chromosome 9, located 3.85 Mb away from a known QTL^7^.

Interestingly, 16 MTAs were identified in genomic regions without previously known loci for eight fruit traits, suggesting discovery of novel QTL. For six of these MTAs, we found candidate genes that are related to fruit development. The GWAS panel used in this study consisted of determinate and semi-determinate tomato accessions that mostly originated from Southern and Western Asian countries. Therefore, this collection is more likely to represent different genetic backgrounds relative to the mapping populations of previous studies^2,7,27,28,32–35^. For example, we found that the fruit height ranged from 25.82 to 86.30 mm with a mean of 52.22 mm, while another population showed 17.46–111.47 mm with a mean of 44.69 mm^28^. Similarly, different phenotypic variations between populations were found for the fruit weight, fruit width, and pericarp thickness. In addition, the fruit shape index used in this study was measured differently from the fruit shape. The first was determined based on the ratio of maximum height and to maximum width using the Tomato Analyser software, while the second was based on 1–9 scales^28^. This distinction could lead to novel QTL identification for the fruit traits in our study.

Two SNP markers showed significant associations with multiple traits, suggesting that corresponding QTL have pleiotropic effects. The SLA773077 marker on chromosome 1 was associated with both the fruit weight and fruit width. The other marker (SLA773357) on chromosome 2 showed associations with the fruit weight, fruit width, and locule number. Since phenotypic correlations between the fruit traits of tomato have been reported in the present and previous studies^28,40,41^, identification of QTL with pleiotropic effects was expected. However, we found no QTL associated with both fruit height and shape index, even though the traits are correlated. In addition, a previous study found several QTL with pleiotropic effects between the fruit weight and fruit height^28^. This result may be due to a small number of MTAs for the fruit height and fruit shape index in our study.

Detection of year or environment specific MTAs commonly occurred in the association mapping studies of tomato fruit traits^7,28,34^. We also found that a few MTAs were detected in all three years. In addition, 14 of 30 MTAs explained < 10% of phenotypic variations for eight fruit traits. Therefore, these MTAs can represent small effect QTL that are easily affected by environmental variations. For marker-assisted selection, large effect QTL has been commonly used in crop breeding programs because this approach was cost-effective and rapid to improve traits of interest. In contrast, MAS has been unsuccessful for complex quantitative traits that are controlled by small effect QTL^42,43^. Genomic selection (GS) has emerged as an alternative to overcome the limitations of MAS for these traits and predicts the breeding values of individuals using a large number of genome-wide markers^43,44^. Recently, it was reported that the use of QTL-associated markers increased the prediction accuracy of GS in several crops^45–47^. In this aspect, the MTAs from our study will be useful for GS in tomato breeding programs.

In conclusion, we reported a total of 30 MTAs for eight fruit traits in a collection of 162 tomato accessions. Of these, 16 MTAs represent potential novel QTL for six fruit traits and in silico analysis found candidate genes in the genomic regions of six MTAs. The resulting SNP markers and candidate genes for these MTAs are a useful resource for further characterization of novel QTL via the fine mapping and gene editing approaches. These MTAs can also be used to investigate a GS method with greater prediction accuracy for fruit traits in tomato. Therefore, our results will benefit the tomato research community by providing an additional tool to breeders for elite cultivar development.

## Materials and methods

### Plant materials and genotyping

The 162 tomato accessions used in this study were derived from a private breeding program and originated from seven countries, including India, China, Turkey, and Israel (Table S1). This collection consisted of determinate and semi-determinate accessions with diverse morphological variations for fruit traits, representing 29 small fruit (< 25 g), 119 medium fruit (25–130 g), and 14 large fruit (> 130 g) tomatoes. For each accession, genomic DNA was isolated using fresh and young leaf tissues from 4-week-old seedlings according to a modified cetyl trimethyl ammonium bromide (CTAB) method^48^. The isolated DNA pellets were resuspended with T1/10E buffer (10 mM Tris-HCl pH 8.0, 0.1 mM EDTA). The quality and quantity of DNA were measured using the NanoDrop^TM^ One spectrophotometer (Thermo Fisher Scientific, Waltham, MA, USA). The final concentration of DNA was adjusted to 50 ng/μL for SNP array-based genotyping.

The collection of 162 tomato accessions was genotyped using the 51 K Axiom^®^ tomato array containing 51,912 SNPs^17^. For this genotyping, 200 ng of genomic DNA from each sample was amplified and randomly fragmented into 25–125 bp using the Axiom^®^ 2.0 reagent kit (Thermo Fisher Scientific, Waltham, MA, USA). The DNA fragments were hybridized to the array in the Affymetrix^®^ GeneTitan system according to the manufacturer’s instructions. The hybridization signals in the form of CEL files were processed using the Affymetirx^®^ Power Tools software package v1.18 for SNP calling. The high-quality SNPs were filtered based on < 10% of missing data, > 5% of minor allele frequency. For the resulting SNPs, missing data were imputed using BEAGLE v5 with default parameter settings^49^.

### Phenotypic evaluation

We evaluated phenotypic variations of fruit weight, fruit width, fruit height, fruit shape index, pericarp thickness, locule number, fruit firmness, and brix over three years (2018–2020) of field trials in the 162 tomato accessions. Plants were first grown in a greenhouse, and 6-7-week-old seedlings were transplanted into plastic-covered fields (high-tunnel) with 30 cm spacing between plants. The field trials were conducted using a randomized complete block design with three replications per genotype and there were four plants per replication. For phenotypic evaluation, fully ripe fruits were harvested from the 2nd to 4th flowering clusters, and 4–10 fruits per replicate for each genotype were used. Image analysis was conducted using the Tomato Analyzer (TA) v4.0 software^50^ for fruit height, fruit width, fruit shape index, locule number, and pericarp thickness. For this analysis, we used ten fruits for small fruit accessions, six fruits for medium fruit accessions, and four fruits for large fruit accessions. Fruits were longitudinally and horizontally cut through the center, placed cut-side down on a scanner, and digitalized according to the user manual of TA^37^. For fruit weight, we used average values of five fruits per replicate. Brix was measured using a PAL-1 refractometer (ATAGO, WA, USA). For fruit firmness, we used the Digital Fruit Firmness Penetrometer (Agriculture Solutions, ME, USA) in 2018 and HPE II Fff (Bareiss, Oberdischingen, Germany) in 2019–2020. The phenotypic data collected in 2018–2020 were independently used for association analysis. An additional data set was also generated by combining those of all three years for seven traits excluding fruit firmness. The combined data set for fruit firmness was based on the two year data (2019 and 2020) due to the use of different penetrometer types. The outliers for the combined data were removed using the IQR method^51^ in R. This data set from multiple years was also used in further association analysis to confirm the result.

### Population structure and association analysis

Population structure in the tomato collection was inferred using the STRUCTURE v.2.3.4 program^52^. The STRUCTURE model used in this study allows for admixture and correlated allele frequencies. To determine the best K (number of clusters), we performed 10 independent simulations for each 10 Ks (1–10) with a burn-in period of 10,000 iterations and a Markov Chain Monte Carlo (MCMC) run length of 10,000 iterations. After the 1st round of analysis, six Ks (4–9) were selected for further simulations with a burn-in period of 20,000 iterations and a MCMC run length of 100,000 iterations. The resulting log-likelihood estimates for the Ks were used to find the best K in the delta K method^30^. A population structure matrix (Q matrix) was then generated using the membership coefficients of 162 tomato accessions based on the best K. In addition, hierarchical clustering was conducted using the R packages. The Nei’s genetic distances^53^ were estimated between tomato accessions using the poppr package^54^ and then hierarchical clustering analysis was conducted with an unweighted pair group method with arithmetic mean (UPGMA).

To identify marker-trait associations (MTAs) for eight fruit traits, we performed association analysis using the multilocus mixed model (MLMM)^18^ implemented in genomic association and prediction integrated tool (GAPIT)^55^. Both Q and kinship matrices were used as covariates to reduce false-positive associations due to population structure and familial relatedness^10^. The kinship matrix was generated using the VanRaden algorithm^56^. Significant MTAs were first detected at *P* < 0.0005. We also used a genome-wide threshold (*P* < 0.00005) that was determined based on the effective number of independent markers, *M*_*e*_^31^. The *M*_*e*_ was estimated using the Genetic Type I Error Calculator (GEC) software (http://pmglab.top/gec/#/) and a genome-wide threshold was calculated with the equation, 0.05/*M*_*e*_. The phenotypic variance explained (PVE) by a significant marker was estimated using the equation in R:

$${{{\mathrm{PVE}}}}\% = \left( {{{{\mathrm{SS}}}}_{{{{\mathrm{sig}}}}{{{\mathrm{.marker}}}}}/\left( {{{{\mathrm{SS}}}}_{{{{\mathrm{all}}}}\,{{{\mathrm{sig}}}}{{{\mathrm{.marker}}}}} + {{{\mathrm{e}}}}} \right)} \right) \times 100$$

where SS is the sum of square and *e* is the residuals from the ANOVA fitted with a linear model incorporating the phenotypic data and all significant markers^57^. Candidate genes for MTAs detected in this study were investigated using the tomato reference genome assembly SL4.0 and the international tomato annotation group (ITAG) 4.0 at the Sol Genomics Network (https://solgenomics.net).

## Supplementary information

Figure S1

Figure S2

Figure S3

Table S1

Table S2
