# Supplementary figures and images for: Genome-wide association study identifies QTL for eight fruit traits in cultivated tomato (Solanum lycopersicum L.)
Source: Hortic Res. 2021 Sep 1;8:203. doi: 10.1038/s41438-021-00638-4 (PMC8408251; doi:10.1038/s41438-021-00638-4)

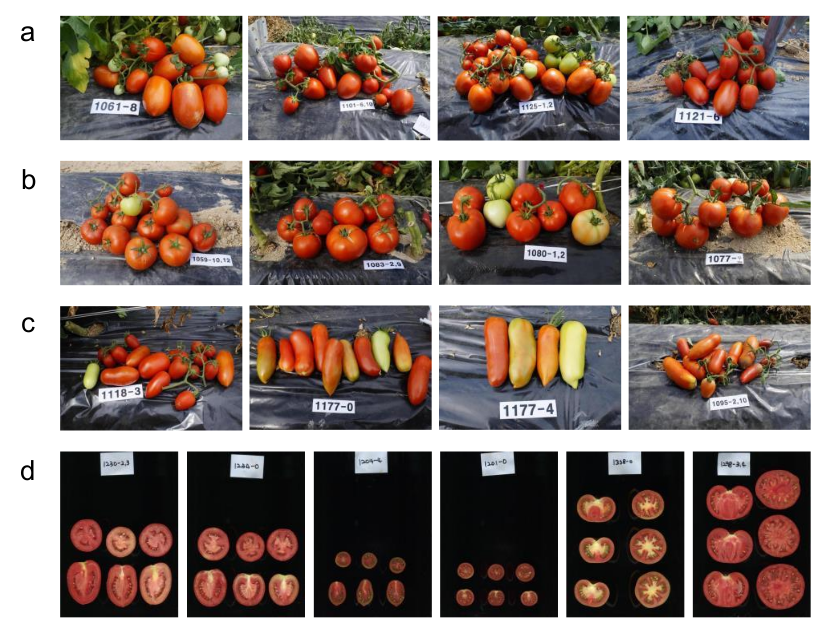

Supplement: Supplementary file 1 — Figure S1 [file 41438_2021_638_MOESM1_ESM.tif]

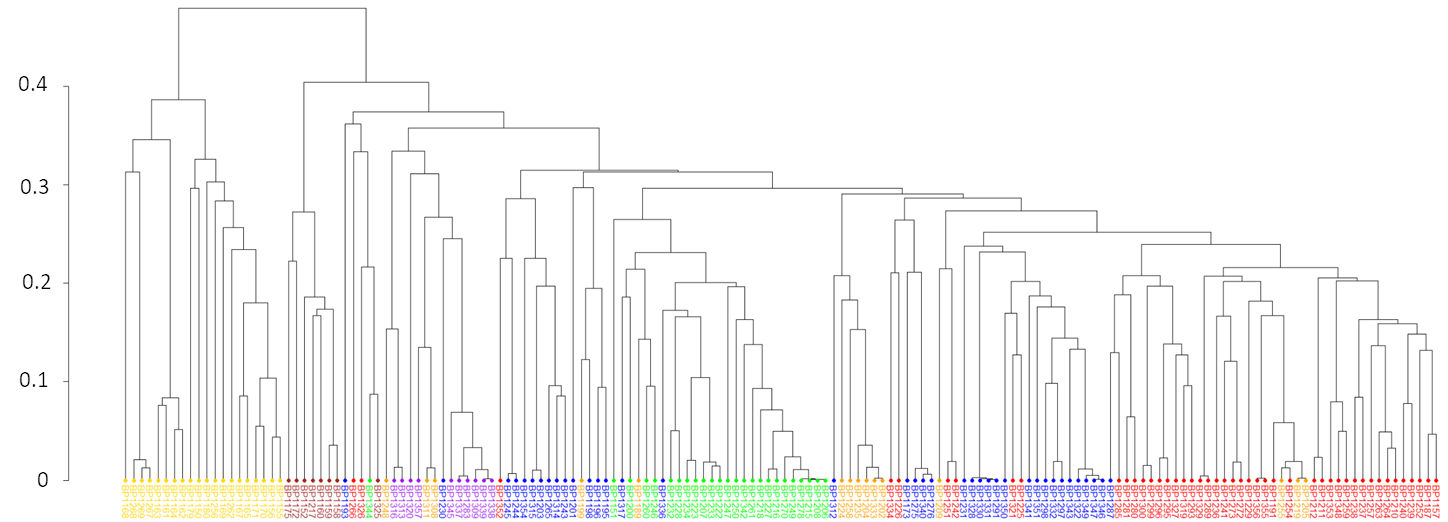

Supplement: Supplementary file 2 — Figure S2 [file 41438_2021_638_MOESM2_ESM.tif]

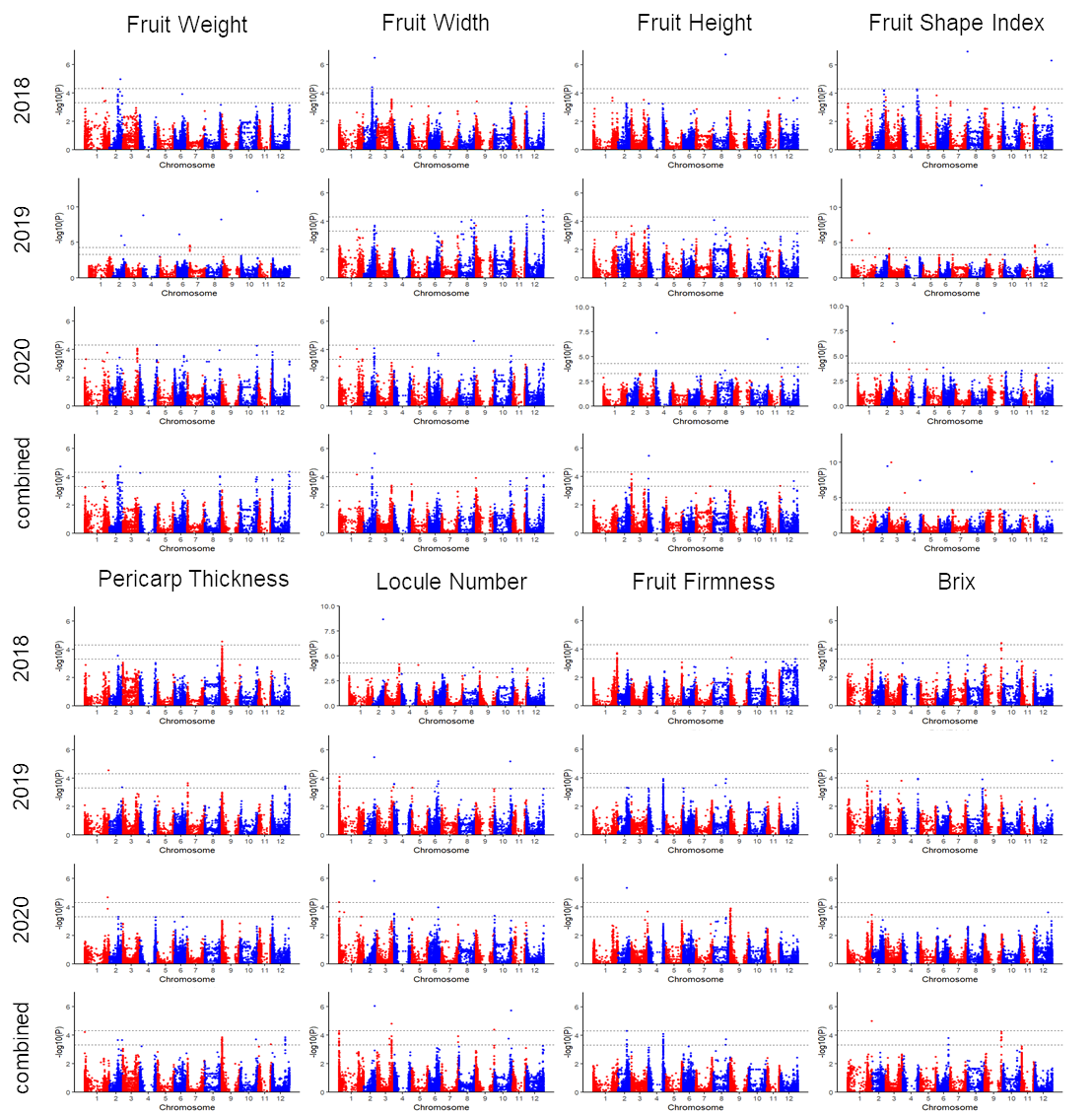

Supplement: Supplementary file 3 — Figure S3 [file 41438_2021_638_MOESM3_ESM.tif]
